# Assessing the Nutritional Quality of Diets of Canadian Adults Using the 2014 Health Canada Surveillance Tool Tier System

**DOI:** 10.3390/nu7125543

**Published:** 2015-12-12

**Authors:** Mahsa Jessri, Stephanie K. Nishi, Mary R. L’Abbé

**Affiliations:** 1 Department of Nutritional Sciences, Faculty of Medicine, University of Toronto, 150 College St.Toronto, ON M5S 3E2, Canada; m.jessri@mail.utoronto.ca; 2 Clinical Nutrition & Risk Factor Modification Center, St. Michael’s Hospital, Toronto, ON M5S 3E2, Canada; s.nishi@mail.utoronto.ca; 3 Department of Nutritional Sciences, Faculty of Medicine, University of Toronto, 150 College St., Toronto, ON M5S 3E2, Canada

**Keywords:** 2014 Health Canada Surveillance Tool Tier system, nutrient profiling, nutritional quality, adults, Canadians

## Abstract

The 2014 Health Canada Surveillance Tool (HCST) was developed to assess adherence of dietary intakes with Canada’s Food Guide. HCST classifies foods into one of four Tiers based on thresholds for sodium, total fat, saturated fat and sugar, with Tier 1 representing the healthiest and Tier 4 foods being the unhealthiest. This study presents the first application of HCST to assess (a) dietary patterns of Canadians; and (b) applicability of this tool as a measure of diet quality among 19,912 adult participants of Canadian Community Health Survey 2.2. Findings indicated that even though most of processed meats and potatoes were Tier 4, the majority of reported foods in general were categorized as Tiers 2 and 3 due to the adjustable lenient criteria used in HCST. Moving from the 1st to the 4th quartile of Tier 4 and “other” foods/beverages, there was a significant trend towards increased calories (1876 kcal *vs.* 2290 kcal) and “harmful” nutrients (e.g., sodium) as well as decreased “beneficial” nutrients. Compliance with the HCST was not associated with lower body mass index. Future nutrient profiling systems need to incorporate both “positive” and “negative” nutrients, an overall score and a wider range of nutrient thresholds to better capture food product differences.

## 1. Introduction

The World Health Organization (WHO), as well as several other international health authorities and regulatory bodies, are developing and supporting the implementation of various “nutrient (or nutritional) profiling” approaches to assess the healthfulness of foods for a wide variety of applications, which may be associated with improved health [1,2,3,4,5,6,7,8]. Nutrient profiling is designed to globally evaluate the healthfulness of food products, based on transparent nutritional composition criteria [1]. Common applications of nutrient profiling include the regulation of front of pack food labeling, health and nutrition claims and food procurement for public institutions (such as schools and hospitals) [1]. With the development in 2014 of Health Canada Surveillance Tool (HCST) [9], the first Canadian nutrient profiling system, there is a potential to broaden the scope of nutrient profiling to assess dietary patterns at a population level. However, this approach has yet to be applied to the dietary intakes of Canadian adults to assess its applicability and relevance.

The HCST aims to assess the food intakes of Canadians relative to the guidance provided by Eating Well with Canada’s Food Guide (EWCFG) [10], based on the classification of foods in the Canadian Nutrient File (CNF) [9,11]. The HCST is the first government-developed nutrient profiling system in Canada and evaluates Canadians’ adherence to EWCFG in terms of amount and type of foods (*i.e.*, number of servings from each food group, and within these, the quality of food choices) [9]. Details regarding this tool have been previously reported by Health Canada [9]. Generally, HCST is a categorical nutrient profiling system that classifies foods within each food group into four Tiers according to their adherence with EWCFG recommendations [9]. The HCST system can then be used to assess Canadians’ eating patterns, based on the proportion of food choices that fall within each Tier [9]. The objectives of the present study were to: (a) assess the quantity and quality of food choices of Canadian adults relative to the HCST Tier system using the Canadian national nutrition survey; and (b) evaluate the applicability and relevance of the HCST as a dietary assessment tool on a population basis.

## 2. Experimental Section

Data from the Canadian Community Health Survey (CCHS) cycle 2.2, was used for this study, which was collected under the authority of the Statistics Act of Canada (2004/5) [12,13]. All data analyses were performed at the Research Data Center of Statistics Canada. The CCHS 2.2 is a multi-stage stratified population-based survey with cluster design, which provides the latest and most complete national nutrition data since the Nutrition Canada Survey conducted in 1972 [12]. The sampling method was designed to be representative of the Canadian population (>98%) in terms of age, sex, geography, and socioeconomic status. The CCHS 2.2 includes cross-sectional nutrition and health data for 35,107 Canadians of all ages from 10 provinces [12]. For the present analysis, we excluded Canadians aged <19 years, pregnant and breastfeeding women, and those with invalid/missing dietary recalls (according to Statistics Canada), leaving a final sample of 19,912 adults. Invalid/missing dietary recalls were defined by Statistics Canada as those with extreme portion sizes and nutrient amounts or with incomplete meals and interviews [14]. Additionally, for evaluation of the applicability and relevance of the Tier system (Objective 2), respondents with missing energy intake, height, weight, and physical activity measures were excluded (final sample: 11,538).

### 2.1. Data Collection and Preparation

Detailed 24-h dietary recall data were obtained using a modified version of the 5-step US Department of Agriculture (USDA) Automated Multiple Pass Method (AMPM) [12,15]. Energy and nutrient composition information for reported foods were derived from Health Canada’s CNF (2001b supplement) [11], which is based on the USDA Nutrient Database for Standard Reference [16] modified to reflect the Canadian food supply and fortifications. Computer-assisted interviews were conducted during all months throughout the year and on all days of the week [12].

Glycemic index (GI) values were determined using the published International GI table values [17,18], which were assigned to each of the Bureau of Nutritional Sciences (BNS) food categories [19] using the procedures proposed by Louie *et al.* and Flood *et al.* [20,21]. Following this method, a BNS group was matched with its corresponding GI; however, if there was no direct match, the GI of a closely-related category was assigned [20,21]. Glycemic load was calculated by multiplying the glycemic index value by the number of grams of carbohydrate then dividing by 100 [17,18]. Energy density of the consumed foods (excluding beverages) was calculated by dividing the total energy from foods (kilocalories) by the total food weight (in grams) [22,23,24]. To reduce extraneous variability and confounding effects, all nutritional analyses were performed in terms of energy intake (using nutrient density approach) [25] and not the absolute amount. In the nutrient density approach, nutrients are expressed per 1000 kcal, and are determined by dividing the amount of the specific nutrient consumed by total energy intake and multiplying by 1000 [25].

As per the procedures of the CCHS 2.2, trained interviewers measured height and weight in person, and body mass index (BMI) was then calculated dividing the weight in kg by the square of height in meters [12]. Respondents were asked about leisure time physical activity during the past 3 months, and socio-demographic and lifestyle behaviours, such as smoking status, and alcohol consumption [12]. All descriptive analyses in this study were stratified by the Institute of Medicine (IOM) Dietary Reference Intakes (DRI) age and sex categories to allow for comparison with national recommendations [26].

### 2.2. Application of the HCST Tier System to Dietary Recalls

#### 2.2.1. Foods Recommended in the EWCFG

The HCST assesses Canadians’ adherence to EWCFG in terms of amount and type of foods consumed [9]. Foods in the CNF were first classified according to the four EWCFG food groups (*i.e.*, Vegetables and Fruits; Grain Products; Milk and Alternatives; Meat and Alternatives); and “other” foods and beverages recommended in EWCFG (*i.e.*, water, and vegetable oil). The four main EWCFG food groups were additionally categorized into 21 subgroups (e.g., subgroups within the Vegetable and Fruits food group: dark green vegetables, deep yellow or orange vegetables, potatoes, other vegetables, vegetable juice and cocktail, fruits other than juice, and fruit juice) [9]. Within each subgroup of the four EWCFG food groups, foods were then categorized into one of four Tiers (Table S1), based on: (1) placement of foods according to EWCFG guidance on total fat, saturated fat, sugars, and sodium (Step 1); and (2) adjustments according to other EWCFG guidance (Step 2) [9]. In general, foods classified as Tier 1 and Tier 2 are considered “foods in line with EWCFG guidance”, Tier 3 foods are “partially in line with EWCFG guidance”, while foods in Tier 4 are described as “foods that are not in line with EWCFG guidance” (Table S1). A detailed description of the food groups and subgroups classified by HCST has been published previously and is briefly explained below [9].

Step 1: Tier 1 foods are those that do not exceed any of the three lower thresholds for total fat (≤3 g/reference amount (RA)), sugars (≤6 g/RA), and sodium (≤140 mg/RA) [9]. The reference amount (RA) provides a standardized basis for a specific food category, and typically is the quantity of a type of food usually eaten by an individual in one sitting [9]. On the other hand, the upper threshold levels of the HCST include: total fat (>10 g/RA), sugars (>19 g/RA), sodium (>360 mg/RA) and saturated fat (>2 g/RA). Foods within Tier 4 exceed at least two upper threshold levels for total fat, sugars, sodium and saturated fat; however, higher exceptions are made for the Milk and Alternatives and Meat and Alternatives food groups which have more inherent saturated fat [9]. Tier 2 and 3 foods fall in between the Tier 1 and Tier 4 foods in terms of healthfulness and nutrient content [9]. Full details of the cut points and applications by food group are shown in Table S1.

Step 2: Additional adjustments were made to reflect other guidance provided by EWCFG, including: consuming at least one dark green and one orange vegetable each day, and having meat alternatives such as beans, lentils and tofu often [9]. Different subgroup codes for orange and green vegetables, as well as for legumes were used for this step after employing the thresholds for total fat, sodium, sugars, and saturated fat [9].

#### 2.2.2. Foods Not Recommended in the EWCFG

Foods that were not among the four main EWCFG food groups and “other” foods and beverages recommended in the EWCFG (*i.e.*, water and vegetable oil) [10], were categorized into the “other” foods and beverages not recommended in the EWCFG, which were further grouped in one of the following subcategories [9]: (a) saturated and/or trans fats and oils (e.g., butter); (b) high fat and/or high sugar foods (e.g., chocolate, candies, sauces, syrups); (c) high-calorie beverages (≥40 kcal/100 g) (e.g., sugar sweetened beverages); (d) low-calorie beverages (<40 kcal/100 g); (e) uncategorized (e.g., dehydrated and condensed soups, ingredients/seasoning and unprepared mixes); (f) meal replacements (e.g., instant breakfast) and supplements (e.g., energy bar); and (g) alcoholic beverages.

Generally, even though it is possible to estimate quantitates equivalent to the Food Guide servings for Tier 4 foods, according to the Health Canada HCST both Tier 4 foods and foods and beverages not included in EWCFG do not have “Food Guide Servings” and both are not in line with the national dietary guidance and therefore can be measured in terms of calories they contribute to the diet. As an example, most cakes, pastries, doughnuts and cookies are categorized as Tier 4 Grain Products, while chocolate and candies are categorized as “other” foods not recommended in Canada’s Food Guide, both of which should be limited.

### 2.3. Definition of Compliance to the HCST Tier System

Since the HCST does not provide a total sum score to represent compliance to the Tier system, we categorized individuals into quartiles based on the percentage of their energy intake from the Tier 4 foods and “other” foods/beverages that are not recommended in the EWCFG [10]. We hypothesized that consumption of higher calories in form of Tier 4 foods and “other” foods and beverages would be associated with higher prevalence of overweight and obesity. Following this classification, individuals with the lowest percentage of energy from Tier 4 and “other” foods/beverages (quartile 1) were labelled as “compliers”, those in the interquartile ranges (quartiles 2 and 3) were “intermediates” and individuals with the highest percentage of energy from Tier 4 and “other” foods/beverages were defined as “non-compliers”. Lifestyle and nutritional characteristics of “compliers”, “intermediates” and “non-compliers” were then compared in order to evaluate the relevance and benefits of adhering to the HCST Tier system.

### 2.4. Identification of Implausible Reporters

Nutritional studies often rely on self-reported dietary intakes, which are prone to dietary under- and over-reporting [27,28]. Recently our group confirmed a widespread prevalence of energy misreporting with higher likelihood among obese individuals and those with chronic diseases (differential misreporting) among participants of the CCHS 2.2 [29]. In addition, we observed higher likelihood of underreporting for foods that are socially undesirable (e.g., high in fat, added sugars and alcohol) (selective misreporting) [29]. We also demonstrated that energy intake misreporting attenuates or reverses the association of dietary exposures with health outcomes; and that adjusting for the misreporting bias is an important consideration in nutritional surveys [29]. In this study, each respondent was classified as under-reporter, plausible reporter or over-reporter by comparing their total Estimated Energy Requirement (EER) and reported energy intake [29,30,31]. IOM factorial equations, established from a meta-analysis of studies measuring EER via doubly-labeled water, were used to calculate EER using participants’ age, sex, BMI, weight, height, and physical activity level (PAL) [26]. Intervals for 4 different levels of physical activity were applied to the data for Canadian adults according to their reported physical activity levels [29,30,31]. Individuals whose EI was less than 70% of their EER were categorized as under-reporters, while those whose EI was more than 142% of their EER were classified as over-reporters (±1 standard deviation) [29,32]. Participants whose EI was between 70% and 142% of their EER were classified as plausible reporters [29,32]. All nutrient profiling analyses in this research were additionally adjusted for the reporting status (under-reporters, plausible reporters, and over-reporters) to account for this systematic bias, as outlined and recommended in our previous study [29].

### 2.5. Statistical Analyses

All statistical analyses were performed using the Statistical Analysis Software (SAS) (version 9.4; SAS Institute Inc., Cary, NC, USA). The bootstrap balanced repeated replication (BBR) method was used to account for the complex multistage survey design in estimation of all standard errors, coefficients of variation and Confidence Intervals (CI) [33,34,35]. All analyses were adjusted for the complex CCHS 2.2 sampling design using appropriate sample weights based on respondent classes with similar socio-demographic characteristics, to maintain a nationally representative sample. Lifestyle and dietary intake characteristics were assessed within age and sex clustered categories by PROC SURVEYREG and PROC SURVEYLOGISTIC for continuous and categorical data, respectively. Group comparison with Tukey post-hoc adjustment was used to evaluate the characteristics of participants classified within DRI age and sex categories. Covariates included in the analysis were age, sex, and dietary recall misreporting status (*i.e.*, under-reporter, plausible reporter, or over-reporter). Results with a two-tailed *p*-value < 0.001 were considered statistically significant.

## 3. Results

### 3.1. Quantity of Food Consumption

Table 1 presents the number of servings from Tier 1 to 3 foods recommended in EWCFG based on the DRI age and sex groups, as well as total number of servings from all Tiers (*i.e.*, 1–4) for comparison, although Tier 4 foods do not have an EWCFG “Food Guide” serving according to Health Canada. Generally the pattern of food consumption choices was consistent across different age groups, even though choices among food groups ranged from healthy foods (Tier 1) to very poor food choices (Tier 4). A few differences, however, were noted. Consumption of vegetables and fruits increased with age (except for a slight decrease among >70 years), especially among women who complied more with the EWCFG recommended number of servings. Within Milk and Alternatives, even though the mean servings were not significantly different between age groups, those over 51 years of age specifically failed to meet recommendations due to their higher requirements. This is even more concerning considering that the recent increase in vitamin D DRI recommendations has not yet been reflected in EWCFG despite Canada’s more Northern latitude [36]. On average, females consumed an equivalent of 0.2 servings of Meat and Alternatives from Tier 4, which is slightly lower compared to their male counterparts at approximately 0.3 servings. As illustrated in Table 2, calories from Tier 4 foods decreased significantly moving from the 19–30 to >70 years old in both males and females (*p* < 0.001). The mean sum of Tier 4 foods and “other” foods and beverages not recommended in EWCFG also decreased with age, comprising 31% and 29% of total calorie intakes in 19–30 years old males and females, compared to 25% and 21% of calories in >70 years old males and females, respectively (*p* < 0.001). The major contributors of “other” foods and beverages were high calorie beverages, high-fat and/or sugar foods, and saturated and/or trans fats and oils.

### 3.2. Quality of Food Consumption

**Figure 1:**
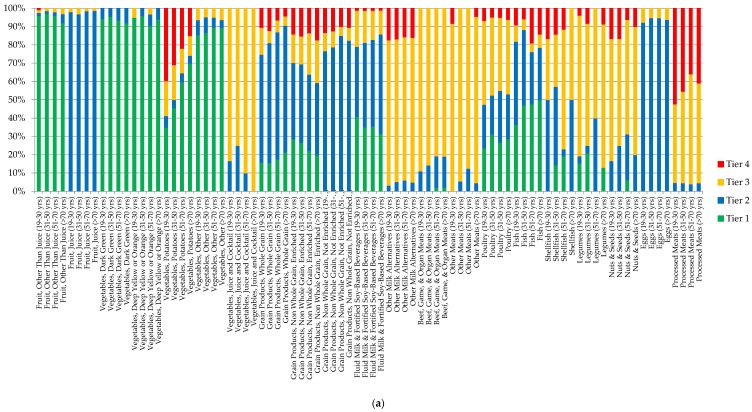

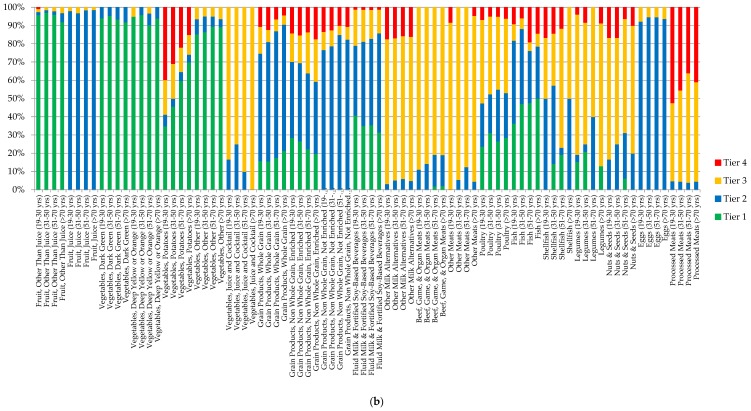
Weighted age-stratified analysis of classification of foods as a percentage of servings based on the 2014 Health Canada Surveillance Tool Tier system among individuals ≥19 years *^,†^ in (**a**) Women and (**b**) Men. * Energy adjusted. ^†^ Tiers are based on Health Canada’s Surveillance Tool [9] and defined generally as follows: Tier 1–3 foods are compliant with EWCFG and Tier 4 foods are not recommended by the EWCFG. Tier 1 foods are foods that do not exceed lower thresholds for total fat, sugars, and sodium; Tier 2 foods do not exceed up to 2 lower thresholds for total fat, sugars or sodium, without exceeding any upper thresholds; for the Vegetables and Fruit and Grain Products food groups Tier 3 are foods that exceed all 3 lower thresholds without exceeding any upper thresholds or exceed only one upper threshold, while Tier 4 foods exceed at least 2 upper thresholds for total fat, saturated fat, sugars, or sodium. Within the Milk and Alternatives and Meat and Alternatives food groups, Tier 3 foods exceed all 3 lower thresholds without exceeding any upper thresholds for total fat, sugars, or sodium (irrespective of saturated fat) or exceed only one of these 3 thresholds or foods that only exceed the upper saturated fat threshold; within these 2 food groups foods that exceed at least 2 upper thresholds for total fat, sugars, or sodium were classified as Tier 4. Where lower thresholds entail: total fat ≤3 g/RA, sugars ≤6 g/RA, and sodium ≤140 mg/RA; and upper thresholds are: total fat >10 g/RA, sugars >19 g/RA, sodium >360 mg/RA, and saturated fat >2 g/RA. Full details are shown in Table S1.

**Figure 2:**
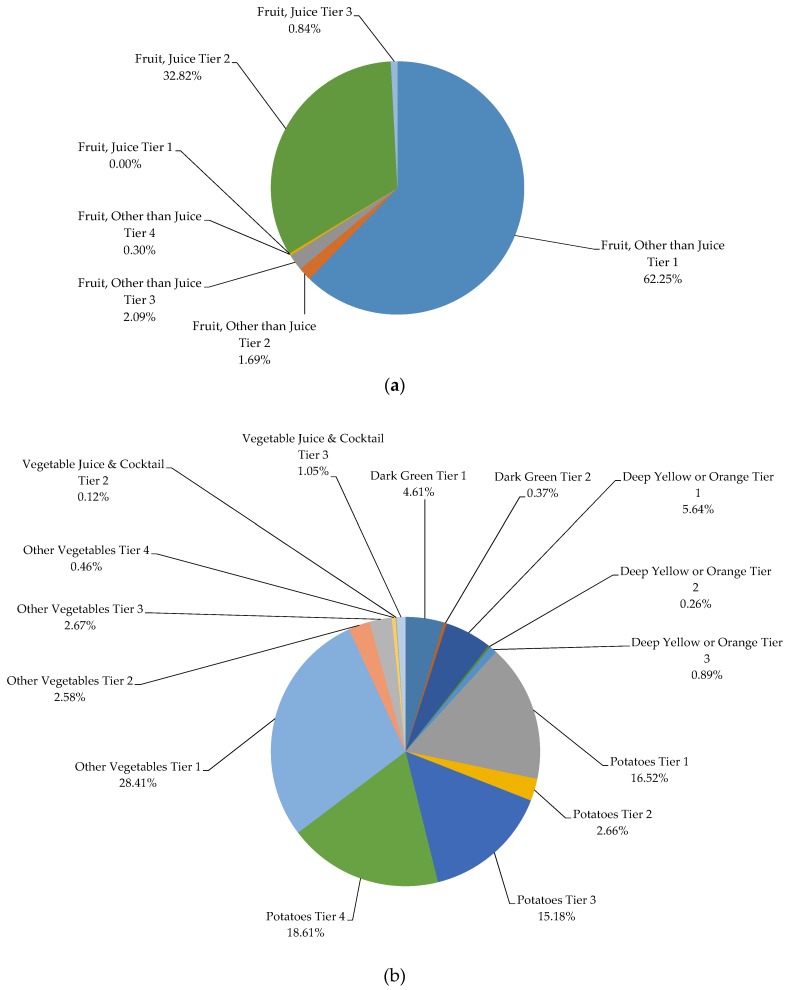

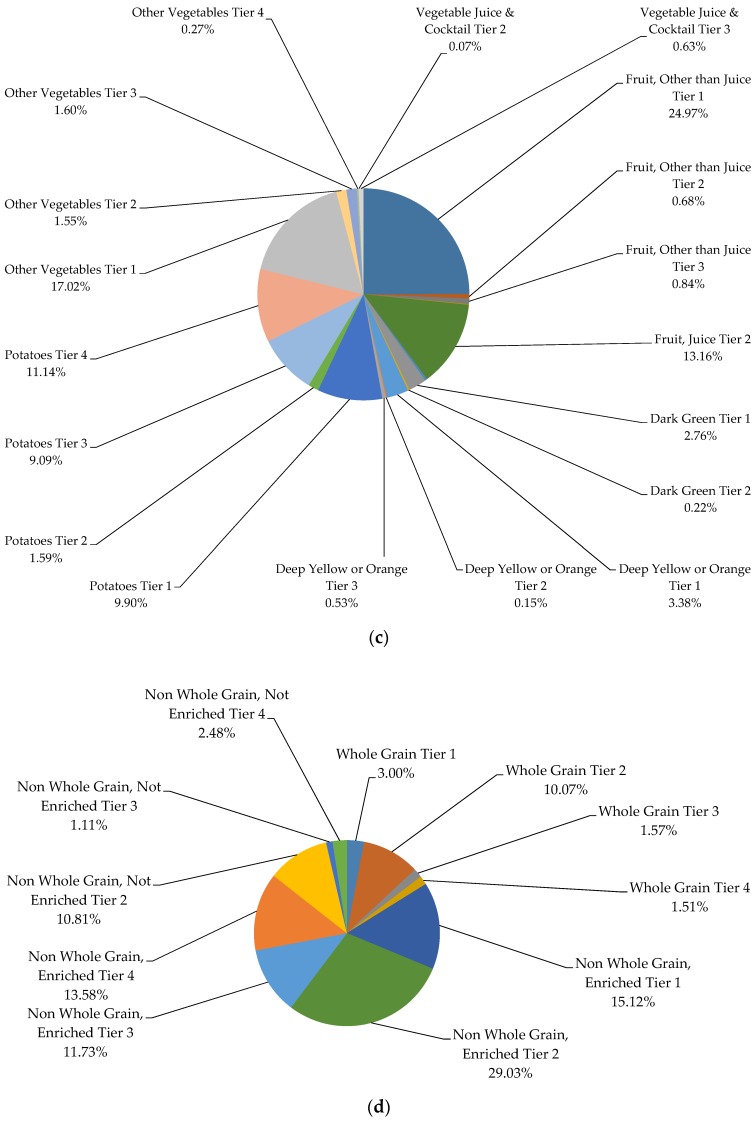

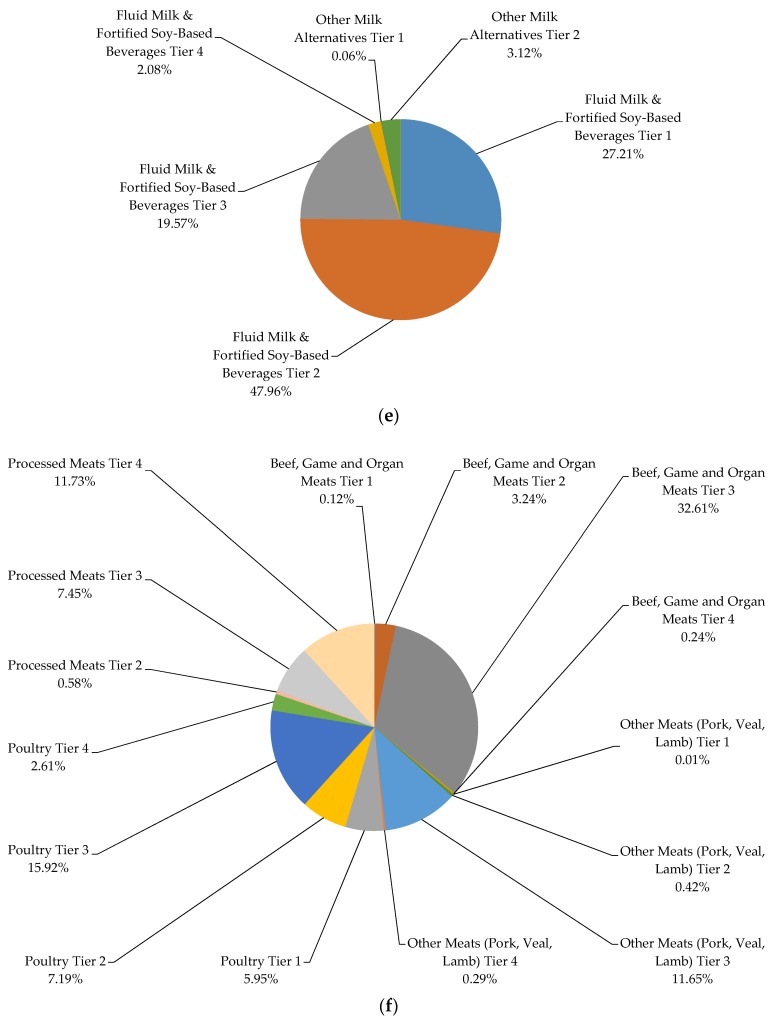

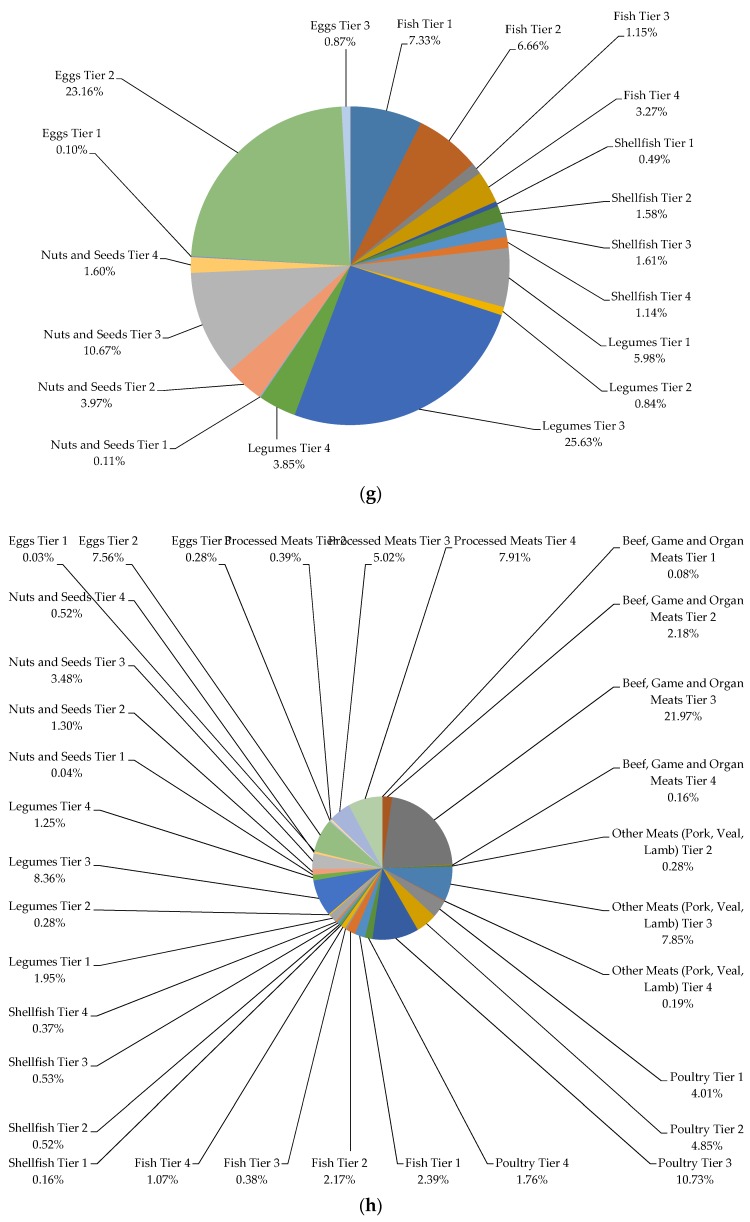
Weighted analysis of percentage of energy intake (kcal) within the (**a**) Fruit Group, (**b**) Vegetable Group; (**c**) Vegetable and Fruit Group; (**d**) Grain Products; (**e**) Milk and Alternatives; (**f**) Meat Group; (**g**) Meat Alternatives Group; (**h**) Meat and Alternatives Group, using the Health Canada Surveillance Tool Tier system among Canadians ≥19 years *. * Tiers are based on Health Canada’s Surveillance Tool [9] and defined generally as follows: Tier 1–3 foods are compliant with EWCFG and Tier 4 foods are not recommended by the EWCFG. Tier 1 foods are foods that do not exceed lower thresholds for total fat, sugars, and sodium; Tier 2 foods do not exceed up to 2 lower thresholds for total fat, sugars or sodium, without exceeding any upper thresholds; for the Vegetables and Fruit and Grain Products food groups. Tier 3 are foods that exceed all 3 lower thresholds without exceeding any upper thresholds or exceed only one upper threshold, while Tier 4 foods exceed at least 2 upper thresholds for total fat, saturated fat, sugars, or sodium. Within the Milk and Alternatives and Meat and Alternatives food groups, Tier 3 foods exceed all 3 lower thresholds without exceeding any upper thresholds for total fat, sugars, or sodium (irrespective of saturated fat) or exceed only one of these 3 thresholds or foods that only exceed the upper saturated fat threshold; within these 2 food groups foods that exceed at least 2 upper thresholds for total fat, sugars, or sodium were classified as Tier 4. Where lower thresholds entail: total fat ≤3 g/RA, sugars ≤6 g/RA, and sodium ≤140 mg/RA; and upper thresholds are: total fat >10 g/RA, sugars >19 g/RA, sodium >360 mg/RA, and saturated fat >2 g/RA. Full details are shown in Table S1.

The highest percentage of servings from Vegetables and Fruits (except for potatoes) consumed by both male and female Canadians were chosen from Tier 1 and 2 classified foods, while the majority of servings from processed meats and potatoes were contributed by Tier 4 foods (Figure 1a,b). The majority of servings from Grain products, Milk and Alternatives, and Meat and Alternatives subgroups were dominated by foods from Tier 2 and Tier 3. When additionally evaluated at the food product level (foods reported in the survey), 20.74% of Fruits and Vegetables, 65.97% of Grain Products, 70.01% of Milk and Alternatives, and 76.35% of Meat and Alternatives food products reported in the CCHS 2.2 were categorized as Tier 2 and 3 (Tables S2,3). In other words, only 24.22% and 6.52% of total food products reported in all 4 food groups met the criteria required to receive the Tier 1 or Tier 4 classification, respectively. In addition, for the products categorized as “other” foods and beverages not included in the EWCFG, the following were the most frequently reported items: ingredients/seasoning and unprepared foods, high fat and/or sugar foods, lower calorie beverages (<40 kcal/100 g), and saturated and/or trans fats and oils. The percentage of calorie intake by Tier categories for each of the EWCFG food groups is presented in Figure 2a–h. As can be seen, within the vegetable group, potatoes (Tiers 1–4) and other vegetables Tier 1 comprised 82% of kilocalories (Figure 2b). About seventy percent of calories from the Grain Products group was contributed by enriched, non-whole grains, with only 16.15% coming from whole grains, which is well below the recommendation for 50% of Grain Products to be whole-grain [10] (Figure 2d). Considering meat products alone, (excluding alternatives), beef, game and organ meats Tier 3 (32.61%), poultry Tier 3 (15.92%), and processed meat Tier 4 (11.73%) made up 60.26% of the total calorie intake from the meat group (Figure 2f).

**Table 1:** Weighted analysis of number of servings from Health Canada’s Eating Well with Canada’s Food Guide (EWCFG) [10] presented based on the 2014 Health Canada’s Surveillance Tool (HCST) Tier system [9] among Canadians ≥19 years *^,†^.

	Men, 19–30 Years	Women, 19–30 Years	Men, 31–50 Years	Women, 31–50 Years	Men, 51–70 Years	Women, 51–70 Years	Men, >70 Years	Women, >70 Years
Food Groups (Servings/Day)	Mean (SEM)	Mean (SEM)	Mean (SEM)	Mean (SEM)	Mean (SEM)	Mean (SEM)	Mean (SEM)	Mean (SEM)
Vegetables and Fruits								
Tiers 1–3	4.35 (0.26)	4.76 (0.19)	4.64 (0.23)	5.12 (0.18)	5.56 (0.21)	5.70 (0.14)	5.37 (0.20)	5.52 (0.15)
Tiers 1–4	4.57 (0.26)	5.01 (0.18)	4.86 (0.23)	5.33 (0.18)	5.71 (0.21)	5.88 (0.14)	5.49 (0.19)	5.67 (0.15)
EWCFG Rec.	8–10	7–8	8–10	7–8	7	7	7	7
Grain Products								
Tiers 1–3	5.37 (0.23)	5.03 (0.16)	5.06 (0.17)	4.83 (0.14)	4.98 (0.16)	4.99 (0.13)	5.23 (0.19)	5.00 (0.12)
Tiers 1–4	5.99 (0.23)	5.81 (0.17)	5.70 (0.18)	5.64 (0.14)	5.55 (0.16)	5.66 (0.13)	5.99 (0.21)	5.82 (0.12)
EWCFG Rec.	8	6–7	8	6–7	7	6	7	6
Milk and Alternatives								
Tiers 1–3	1.34 (0.10)	1.62 (0.08)	1.17 (0.08) ^b^	1.58 (0.07) ^b^	1.23 (0.08)	1.46 (0.06)	1.55 (0.13)	1.59 (0.06)
Tiers 1–4	1.51 (0.10)	1.79 (0.08)	1.34 (0.08) ^b^	1.75 (0.07) ^b^	1.37 (0.08)	1.59 (0.06)	1.64 (0.13)	1.72 (0.06)
EWCFG Rec.	2	2	2	2	3	3	3	3
Meat and Alternatives								
Tiers 1–3	1.86 (0.15)	1.70 (0.10)	2.35 (0.11)	2.01 (0.09)	2.40 (0.11)	2.19 (0.09)	2.10 (0.11)	2.01 (0.08)
Tiers 1–4	2.12 (0.14) ^a^	1.88 (0.09) ^a^	2.60 (0.10) ^b^	2.20 (0.09) ^b^	2.65 (0.10)	2.40 (0.09)	2.36 (0.10)	2.21 (0.08)
EWCFG Rec.	3	2	3	2	3	2	3	2

Rec.: Recommendation; SEM: Standard Error of Mean; * Energy adjusted; ^†^ Tiers are based on Health Canada’s Surveillance Tool [9] and defined generally as follows: Tier 1–3 foods are compliant with EWCFG and Tier 4 foods are not recommended by the EWCFG. Tier 1 foods are foods that do not exceed lower thresholds for total fat, sugars, and sodium; Tier 2 foods do not exceed up to 2 lower thresholds for total fat, sugars or sodium, without exceeding any upper thresholds; for the Vegetables and Fruit and Grain Products food groups Tier 3 are foods that exceed all 3 lower thresholds without exceeding any upper thresholds or exceed only one upper threshold, while Tier 4 foods exceed at least 2 upper thresholds for total fat, saturated fat, sugars, or sodium. Within the Milk and Alternatives and Meat and Alternatives food groups, Tier 3 foods exceed all 3 lower thresholds without exceeding any upper thresholds for total fat, sugars, or sodium (irrespective of saturated fat) or exceed only one of these 3 thresholds or foods that only exceed the upper saturated fat threshold; within these 2 food groups foods that exceed at least 2 upper thresholds for total fat, sugars, or sodium were classified as Tier 4. Where lower thresholds entail: total fat ≤3 g/RA, sugars ≤6 g/RA, and sodium ≤140 mg/RA; and upper thresholds are: total fat >10 g/RA, sugars >19 g/RA, sodium >360 mg/RA, and saturated fat >2 g/RA. Full details are shown in Table S1; ^a^ Comparison significantly different between 19 and 30 years old males and females, based on Tukey multiple comparison test (*p* < 0.001); ^b^ Comparison significantly different between 31 and 50 years old males and females, based on Tukey’s multiple comparison test (*p* < 0.001).

**Table 2:** Weighted analysis of energy contribution from Tiers 1–3 foods (compliant with Eating Well with Canada’s Food Guide (EWCFG)) [9] and Tier 4 and “other” foods and beverages not included in the EWCFG [9] among Canadian adults (≥19 years) *.

	Men, 19–30 Years	Women, 19–30 Years	Men, 31–50 Years	Women, 31–50 Years	Men, 51–70 Years	Women, 51–70 Years	Men, >70 Years	Women, >70 Years
Variable (kcal/Day)	Mean (SEM)	Mean (SEM)	Mean (SEM)	Mean (SEM)	Mean (SEM)	Mean (SEM)	Mean (SEM)	Mean (SEM)
Tiers 1 + 2 + 3	1539 (34)	1089 (24)	1468 (29)	1103 (16)	1353 (20)	1086 (17)	1217 (23)	1014 (16)
Tier 4	269 (15)	175 (10)	240 (12)	161 (9)	178 (10)	125 (7)	164 (10)	114 (6)
**Other Foods/Beverages**								
Alcoholic beverages	155 (12)	63 (9)	127 (8)	63 (6)	124 (8)	42 (3)	62 (6)	22 (2)
Beverages, higher calorie (≥40 kcal/100g)	171 (8)	103 (6)	108 (6)	61 (4)	58 (4)	42 (4)	30 (3)	26 (2)
Beverages, lower calorie (<40 kcal/100g)	30 (3)	26 (2)	29 (2)	26 (2)	23 (2)	19 (1)	16 (1)	15 (1)
High fat and/or sugar foods	153 (8)	130 (7)	167 (9)	124 (7)	123 (5)	105 (5)	121 (7)	87 (5)
Meal replacements	7 (2)	6 (2)	4 (1)	5 (1)	2 (1)	4 (1)	0 (0)	1 (0)
Saturated and/or trans fats and oils	74 (5)	54 (4)	80 (5)	59 (3)	87 (4)	62 (3)	78 (4)	64 (4)
Supplements	4 (2)	1 (0)	3 (2)	1 (0)	1 (0)	2 (1)	2 (1)	3 (1)
Uncategorized (ingredients/seasonings and unprepared foods)	22 (3)	15 (1)	20 (2)	17 (1)	18 (1)	16 (1)	15 (1)	14 (1)
Unsaturated fats and oils	83 (5)	57 (5)	71 (4)	62 (3)	70 (4)	55 (2)	51 (3)	47 (3)
Total energy from Tier 4 and “other” foods/beverages (kcal/day)	874 (27)	567 (19)	771 (21)	510 (16)	611 (17)	411 (11)	487 (18)	342 (11)
Total energy from Tier 4 and “other” foods/beverages (%)	31 (1)	29 (1)	30 (1)	27 (1)	27 (1)	23 (0)	25 (1)	21 (0)

SEM: Standard Error of Mean; * “Other” foods/beverages are not part of the Tier system and include “other” food and beverages not in the groups of Eating Well with Canada’s Food Guide [9].

### 3.3. Diets High in Calories from Tier 4 and “Other” Foods/Beverages Are Not Associated with Obesity

As presented in Table 3, individuals in quartile 1 of calories from Tier 4 and “other” foods and beverages (compliers) were more likely to be older (*p*-trend < 0.0001), female (*p*-trend: 0.0175), physically active (*p*-trend: 0.0342), and non-smokers (*p*-trend < 0.0001) compared to the intermediate- and non-compliers.

However, there was no significant trend observed between more compliance to the HCST recommendations and BMI in the present study (*p*-trend: 0.3214). Additional regression analysis adjusted for age and sex did not reveal any significant associations (odds ratio for quartile 4 *vs.* quartile 1: 1.058 (0.799–1.397); quartile 3 *vs.* quartile 1: 1.047 (0.792–1.384); quartile 2 *vs.* quartile 1: 0.872 (0.646–1.176) (*p*-trend: 0.7053) (Table S4).

### 3.4. Diets High in Calories from Tier 4 and “Other” Foods/Beverages Are Associated with a Lower Nutrient Dense Diet

**Figure 3:**
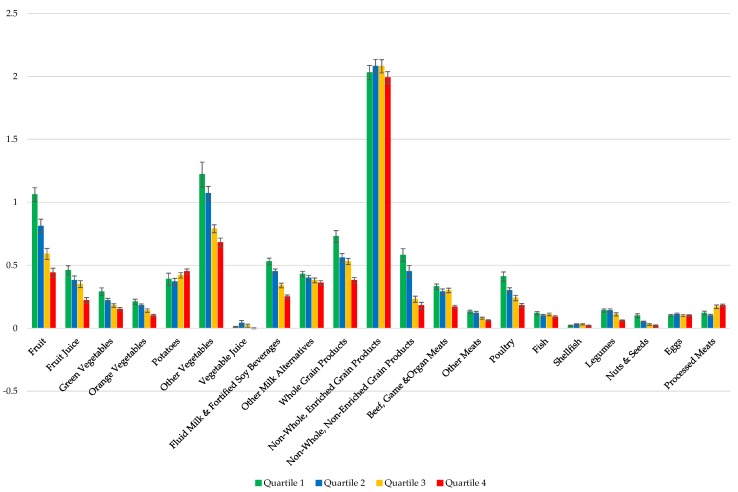
Implementation of 2014 Health Canada Surveillance Tool Tier system to the dietary intakes of Canadian adults (≥19 years) in a weighted analysis to assess the number of serving from each of the Eating Well with Canada’s Food Guide subgroups per 1000 kcal [25]. Dietary profiles of compliers (Quartile 1) *, intermediates (Quartiles 2 and 3) ^†^, and non-compliers (Quartile 4) ^‡^ are compared ^§,‖^ NS, Not significant; * The 25% of individuals with the lowest percentage of energy from Tier 4 and “other” foods and beverages; ^†^ The individuals in the interquartile range for energy intakes from Tier 4 and “other” foods and beverages; ^‡^ The 25% of individuals with the highest percentage of energy from Tier 4 and “other” foods and beverages; ^§^ Adjusted for age, sex, and misreporting status (under-reporters, plausible-, and over-reporters); ^‖^ Quartiles are based upon percentage of energy from all Tier 4 foods based on 2014 Health Canada’s Surveillance Tool Tier system plus “other” foods and beverages not recommended in the Eating Well with Canada’s Food Guide.

The mean servings of EWCFG food subgroups per 1000 kcal among compliers (Q1), intermediate compliers (Q2 and Q3), and non-compliers is presented in Figure 3. After adjusting for age, sex and misreporting status, individuals in the highest quartile category of the percentage of energy from Tier 4 and “other” foods/beverages (non-compliers) consumed significantly higher servings of processed meat per 1000 kcal (0.18 ± 0.011) compared to those in the lowest quartile (0.12 ± 0.015) (*p*-trend < 0.0001). Similarly, mean servings per 1000 kcal of potatoes was higher among non-compliers, even though the *p*-trend did not reach the statistical significance level (*p*-trend: 0.1402). Generally, the mean servings of all Fruit, Vegetable (excluding potatoes), Milk and Alternatives, Grains Products (except for refined enriched grains), and Meat and Alternatives (excluding processed meats, fish, shellfish, egg) subgroups per 1000 kcal were significantly higher in the complier group compared to the non-compliers (Figure 3).

The nutrient intakes of compliers, intermediates and non-compliers reported in terms of energy density [25] and adjusted for age, sex and misreporting are presented in Table 4. Generally, compliers consumed significantly less energy (on average 415 kcal/day) compared to non-compliers (*p*-trend: <0.0001). Similarly, there was a significant trend towards increasing the percentage energy from fat, saturated fat, mono-unsaturated fat, poly-unsaturated fat, added sugars, and alcohol intake with less compliance to EWCFG guidance (*p* < 0.0001). In addition, the intakes of fiber, protein, vitamin A, vitamin D, all B-vitamins, and vitamin C decreased significantly moving from quartile 1 to 4, indicating that those consuming the most energy from Tier 4 and “other” foods and beverages (*i.e.*, non-compliers) have a less nutrient dense diet. Consumption of minerals, including calcium, phosphorus, potassium, magnesium, iron, and zinc, was significantly lower in the non-compliers compared to the intermediate and compliers (*p* < 0.0001). Similarly, glycemic index, and energy density were significantly higher in the non-complier group compared to the intermediates and compliers (*p*-trend < 0.0001).

**Table 3:** Weighted analysis of characteristics of compliers, intermediates, and non-compliers based on the percentage of energy from Tier 4 foods and “other” foods/beverages among Canadian adults (≥19 years) *^,†^.

	Compliers (Q1) ^‡^ ≤19.42% Energy	Intermediates (Q2) ^§^ 19.42%–31.78% Energy	Intermediates (Q3) ^§^ 31.78%–45.73% Energy	Non–compliers (Q4) ^‖^ >45.73% Energy	
Characteristics	Mean (SEM)	Mean (SEM)	Mean (SEM)	Mean (SEM)	*p*-Trend
Age (years)	49.82 (0.57)	47.38 (0.72)	46.05 (0.54)	42.57 (0.49)	<0.0001
Sex (%)					
Males	44.10 (3.57)	48.84 (3.73)	51.76 (2.47)	53.48 (2.24)	
Females	55.90 (3.57)	51.16 (3.73)	48.24 (2.47)	46.52 (2.24)	0.0175
BMI (kg/m^2^)	27.62 (0.28)	27.18 (0.19)	27.42 (0.19)	27.69 (0.21)	0.3214
Misreporting Status (%)					
Under Reporters	42.87 (2.03)	34.35 (2.54)	26.87 (1.52)	22.66 (1.64)	
Over Reporters	9.02 (1.39)	7.97 (1.05)	9.94 (1.21)	14.29 (1.49)	<0.0001
Physical Activity (%)					
Inactive	55.86 (2.48)	56.83 (1.96)	57.54 (1.85)	62.93 (1.84)	
Active	18.97 (1.53)	18.86 (1.41)	15.80 (1.24)	15.87 (1.28)	0.0342
Smoking Status (%)					
Daily Smoker	13.07 (1.32)	14.56 (1.17)	24.61 (1.76)	30.33 (1.68)	
Never Smoked	57.68 (1.97)	48.33 (2.07)	41.89 (1.88)	34.10 (1.53)	<0.0001

SEM: Standard Error of Mean; * Adjusted for age and sex; ^†^ Quartiles are based upon percentage of energy from all Tier 4 foods based on 2014 Health Canada’s Surveillance Tool Tier system plus “other” foods and beverages not recommended in the Eating Well with Canada’s Food Guide; ^‡^ The 25% of individuals with the lowest percentage of energy from Tier 4 and “other” foods and beverages; ^§^ The individuals in the interquartile range for energy intakes from Tier 4 and “other” foods and beverages; ^‖^ The 25% of individuals with the highest percentage of energy from Tier 4 and “other” foods and beverages.

**Table 4:** Weighted analysis of nutrient intakes (density approach) [25] by compliers, intermediates, and non-compliers based on the percentage of energy consumed from Tier 4 foods and “other” foods/beverages among Canadian adults (≥19 years), adjusted for age, sex, and misreporting status (under-reporter, plausible-, and over-reporters) *.

	Compliers (Q1) ^†^ ≤19.42% Energy	Intermediates (Q2) ^‡^ 19.42%–31.78% Energy	Intermediates (Q3) ^‡^ 31.78%–45.73% Energy	Non–compliers (Q4) ^§^ >45.73% Energy	*p*-Trend
Nutrients	Mean (SEM)	Mean (SEM)	Mean (SEM)	Mean (SEM)	
Energy (kcal/day)	2355 (28)	2426 (24)	2427 (22)	2478 (30)	<0.0001
Fat (%Energy)	28.90 (0.43)	31.70 (0.34)	33.91 (0.32)	33.73 (0.39)	<0.0001
Saturated fat (%Energy)	9.02 (0.15)	10.02 (0.16)	11.23 (0.18)	11.29 (0.19)	<0.0001
Monounsaturated fat (%Energy)	11.37 (0.22)	12.74 (0.18)	13.59 (0.15)	13.64 (0.19)	<0.0001
Polyunsaturated fat (%Energy)	5.35 (0.13)	5.76 (0.10)	5.96 (0.10)	5.91 (0.11)	0.00
Carbohydrates (%Energy)	50.83 (0.56)	48.77 (0.55)	47.13 (0.46)	47.29 (0.47)	<0.0001
Added sugar (%Energy)	5.45 (0.23)	7.71 (0.31)	10.40 (0.29)	14.00 (0.36)	<0.0001
Dietary fiber (g/1000 kcal)	10.99 (0.30)	9.37 (0.16)	7.99 (0.13)	6.86 (0.11)	<0.0001
Protein (%Energy)	19.63 (0.32)	17.46 (0.30)	15.78 (0.22)	12.82 (0.16)	<0.0001
Alcohol (%Energy)	0.64 (0.13)	2.07 (0.13)	3.17 (0.24)	6.16 (0.41)	<0.0001
Vitamin A (RE/1000 kcal)	454.94 (37.78)	377.34 (37.78)	339.23 (11.48)	287.96 (10.65)	<0.0001
Vitamin D (ug/1000 kcal)	3.23 (0.16)	3.18 (0.15)	2.84 (0.19)	2.45 (0.16)	<0.0001
Thiamin (mg/1000 kcal)	1.01 (0.02)	0.91 (0.01)	0.81 (0.01)	0.66 (0.01)	<0.0001
Riboflavin (mg/1000 kcal)	1.09 (0.02)	0.99 (0.01)	0.90 (0.01)	0.80 (0.01)	<0.0001
Niacin (NE/1000 kcal)	23.09 (0.40)	20.45 (0.27)	18.66 (0.26)	16.03 (0.31)	<0.0001
Vitamin B6 (ug/1000 kcal)	1.18 (0.02)	0.98 (0.01)	0.86 (0.02)	0.71 (0.01)	<0.0001
Folate (ug/1000 kcal)	140.90 (4.18)	126.67 (2.72)	111.50 (2.16)	94.84 (1.86)	<0.0001
Vitamin B12 (ug/1000 kcal)	2.60 (0.17)	2.44 (0.16)	2.03 (0.06)	1.69 (0.06)	<0.0001
Vitamin C (mg/1000 kcal)	77.08 (2.35)	67.80 (2.22)	59.87 (1.90)	45.63 (1.76)	<0.0001
Calcium (mg/1000 kcal)	480.37 (9.83)	437.20 (8.22)	392.96 (6.56)	349.48 (6.24)	<0.0001
Phosphorous (mg/1000 kcal)	772.27 (8.81)	691.04 (7.52)	622.08 (7.17)	547.76 (6.62)	<0.0001
Potassium (mg/1000 kcal)	1855.09 (27.90)	1644.51 (16.61)	1477.51 (16.16)	1295.64 (18.68)	<0.0001
Sodium (mg/1000 kcal)	1536.45 (30.10)	1523.69 (25.57)	1584.82 (24.92)	1510.05 (35.78)	0.11
Magnesium (mg/1000 kcal)	194.68 (3.53)	173.05 (1.99)	152.38 (1.57)	140.98 (4.48)	<.0001
Iron (mg/1000 kcal)	8.04 (0.12)	7.38 (0.10)	6.75 (0.08)	5.75 (0.07)	<.0001
Zinc (mg/1000 kcal)	6.63 (0.10)	5.89 (0.13)	5.37 (0.09)	4.33 (0.07)	<0.0001
Glycemic Index	51.03 (0.40)	52.40 (0.32)	53.61 (0.32)	53.56 (0.35)	<0.0001
Glycemic Load	151.26 (3.12)	156.99 (2.72)	154.48 (2.48)	159.37 (2.94)	0.07
Energy Density (kcal/g)	1.55 (0.02)	1.71 (0.02)	1.91 (0.02)	2.15 (0.02)	<0.0001

* Quartiles are based upon percentage of energy from all Tier 4 foods based on 2014 Health Canada’s Surveillance Tool Tier system plus “other” foods and beverages not recommended in the Eating Well with Canada’s Food Guide; ^†^ The 25% of individuals with the lowest percentage of energy from Tier 4 and “other” foods and beverages; ^‡^ The individuals in the interquartile range for energy intakes from Tier 4 and “other” foods and beverages; ^§^ The 25% of individuals with the highest percentage of energy from Tier 4 and “other” foods and beverages.

## 4. Discussion

The present study provides the first assessment of the 2014 HCST Tier system based upon national Canadian nutrition data. Assessment of eating habits using this nutrient profiling system revealed that the quality and quantity of Canadian adults’ eating patterns are not meeting Health Canada’s recommendations. Specifically, Canadian adults are not meeting Health Canada’s recommended number of food group servings, and there is a high prevalence of consumption of Tier 4 classified foods among this population, especially Tier 4 processed meats and potatoes. Importantly, one-third of daily calories were consumed from Tier 4 and “other” food/beverage sources not recommended in the EWCFG. Using this nutrient profiling system, the majority of food choices of Canadians (except for vegetables and processed meats) were categorized as either Tiers 2 or 3, despite the large variation among the food items reported. This lack of specificity questions the validity of HCST and discriminative ability of its thresholds for use to evaluate national eating patterns. These findings may also justify the lack of significant associations between adherence to HCST and obesity among CCHS 2.2 participants. However, closer compliance to HCST system indicated increased probability of meeting DRI nutrient recommendations, which is expected since the HCST was developed to evaluate adherence to EWCFG, which itself is modeled based on achieving DRI recommendations [10,36]. Similarly, since HCST is in line with EWCFG, it does not address recommendations for obesity and chronic disease prevention [36,37], which may also explain lack of significant associations between HSCT compliance and obesity risk in this study.

In 2015, the World Health Organization (WHO) published a report developing a common nutrient profiling model for Europe based on review of existing nutrient profiling models [2], including those published by the governments of the United Kingdom, Australia and New Zealand, and the United States [4,8]. The WHO model consists of 17 food categories with pre-defined thresholds for the contents of energy, total fat, saturated fat, total sugars, added sugars, and sodium to help authorities identify unhealthy foods [2]. Compared to the HCST with 9 food categories, the WHO model consists of 17 food categories, even though both systems use pre-defined thresholds for classifying different foods [2,9]. The HCST food categories contain a large variation within each Tier subgroup due to the broad discrete definitions used for defining the thresholds for Tiers 1 and 4, and the lenient adjustable criteria used to categorize foods into Tiers 2 and 3 [9]. In the present study, a consequence of HCST limitations was categorization of the majority of foods into Tiers 2 and 3, except for fruits and vegetables where the majority were classified as Tier 1 and 2 as well as processed meats, despite large product differences. As an example, foods categorized by the BNS Food Group Descriptions [19] as “jello, dessert toppings and pudding mixes-commercial” could fall within both Tier 2 and Tier 3 of the HCST. In addition, the limited range of thresholds in HCST results in a small percentage of products to be categorized as Tiers 1 or 4, especially in sub-groups such as fluid milk and fortified soy-based beverages and fruit juice, with more similarities among products. This is in contrast to the United Kingdom’s Ofcom Model, which calculates a total score for food items based on the total points for “negative” nutrients (energy, total sugar, saturated fat, and sodium) subtracted by points obtained for “positive” nutrients (fruits, vegetables and nuts, fiber, and protein) [4]. Based upon the Ofcom model, the Nutrient Profiling Scoring Criterion (NPSC), developed by Food Standards Australia New Zealand (FSANZ), not only considers sodium, saturated fat, and sugar content of foods, but it also accounts for ingredients such as dietary fiber, protein and fruit and vegetables and calculates a total nutrient profiling score for a food [8].

Since 2010, the United States NuVal Nutritional Scoring System has been used on the basis of the Overall Nutritional Quality Index (ONQI) algorithm [5,6,7]. The ONQI incorporates over 30 nutrients and food properties, in addition to weighting coefficients (energy density, glycemic load, protein quality, and fat quality) representing epidemiologic associations between nutrients and health outcomes [5]. ONQI summarizes comprehensive nutritional information into a single score ranging from 1 to 100 based on their relative nutrition and healthfulness [5]. Adherence to the ONQI has previously been associated with lower risk of total chronic diseases and total mortality during over 20 years of follow-up, although the lack of transparency of this tool has remained controversial [5,38].

In the present study, the HCST was able to distinguish the diet quality of compliers, intermediate compliers, and non-compliers, which is in line with the findings of previous research using other indexes [39,40,41]. Favorable diet quality in terms of lower consumption of Tier 4 and “other” foods/beverages was associated with higher intakes of vitamins and minerals, and lower intakes of energy, fats, added sugars, alcohol, glycemic index, and energy density, even though these nutritional components were not considered in the quartile categorization of individuals.

In addition, our results confirm previous research indicating that older, female, physically active, and non-smoker individuals have healthier dietary quality, which is also an indication of the face validity of HCST in the Canadian population [39,42,43,44,45]. In particular, lower diet quality was seen among smokers, who have been previously shown to be less physically active, and have high alcohol intakes and low consumption of fruits and vegetables [46,47], which may be due to taste modifications, dysregulation of appetite, and unhealthy lifestyle among this group [47].

In this research, we failed to observe a significant association between adherence to a nutrient profiling system and BMI, which is in line with some previous studies [42,48]. This lack of association may be explained by the focus of the EWCFG and HCST on meeting the DRI nutrient requirements rather than disease prevention [36]. Our group recently published a critical analysis of the EWCFG concluding that adherence to the EWCFG does not necessarily guarantee a reduced risk of obesity or other chronic diseases [36], since the EWCFG has been modeled to strictly meet the DRI nutrient recommendations. Even though some *a priori* diet quality indexes have been negatively associated with the risk of obesity (including healthy eating index and dietary quality index among CCHS 2.2 participants [49]), others have found neutral [48] or even inverse [50] associations. These inconsistent results may also be related to the cross-sectional nature of studies, or the observation that overweight and obese individuals are more likely to watch their nutritional intake or to be dieting [51,52].

To our knowledge, this is the first study to investigate the application of a nutrient profiling system in characterizing the diet quality of Canadians, which is of high public health importance. Nutrient profiling systems as well as dietary quality scores aim to evaluate overall diet quality of individuals using available scientific evidence about the role of diet in health promotion [53]. Considering the correlation between foods and nutrients and totality of diet are important advantages of using diet quality indexes and nutrient profiling systems [53]. Strengths of our study include the use of a large nationally-representative sample, including several covariates, having measured anthropometry, and use of the USDA AMPM which minimized misreporting bias as a result of missing items or eating occasion.

This study is not without its limitations. One limitation is the day-to-day variation (random non-differential error) associated with 24-h dietary recalls. Another disadvantage common among all diet quality index analyses is the subjectivity surrounding the selection of nutritional components, threshold values, and scoring criteria [54]. The major limitation of HCST is the strict focus on 4 “negative” nutrients (total fats, saturated fat, sodium, and sugars) and lack of calculation of a total dietary score, which prevents direct comparisons across groups. Finally, owing to the cross-sectional design of the national Canadian nutrition survey, the causal inference is limited.

## 5. Conclusions

The 2014 HCST Tier system proves to have good validity for characterizing dietary intakes of Canadian adults and therefore could be used for public health initiatives to ensure adherence to EWCFG recommendations. However, it must be noted that this system was not a good indicator of obesity, which is in line with previous studies that have criticized the overly focus of the EWCFG on meeting DRI nutrient requirements, rather than chronic disease prevention [36]. In light of recent improvements and updates in dietary guideline (e.g., Scientific Report of the Dietary Guidelines for Americans 2015 [55]) and strong evidence for the role of nutrition in the prevention of chronic diseases, revising the EWCFG model to reflect these may form a more appropriate platform for development of future Canadian nutrient profiling systems [36]. Future Canadian nutrient profiling systems should also take an approach similar to that taken by the United Kingdom Ofcom model, FSANZ, and/or the ONQI, to include a more complex algorithm that incorporates several “positive” and “negative” nutrients, provide a total summative score, and consider associations with chronic diseases risk.

## Supplementary Materials

Supplementary materials can be accessed at: http://www.mdpi.com/2072-6643/7/12/5543/s1.
