# Correction: Jessri, M.; et al. Assessing the Nutritional Quality of Diets of Canadian Adults Using the 2014 Health Canada Surveillance Tool Tier System. *Nutrients* 2015, *7*, 5543

**DOI:** 10.3390/nu9010058

**Published:** 2017-01-12

**Authors:** Mahsa Jessri, Stephanie K. Nishi, Mary R. L’Abbé

**Affiliations:** 1 Department of Nutritional Sciences, Faculty of Medicine, University of Toronto, 150 College St., Toronto, ON M5S 3E2, Canada; m.jessri@mail.utoronto.ca; 2 Clinical Nutrition & Risk Factor Modification Center, St. Michael’s Hospital, Toronto, ON M5S 3E2, Canada; s.nishi@mail.utoronto.ca; 3 Department of Nutritional Sciences, Faculty of Medicine, University of Toronto, 150 College St., Toronto, ON M5S 3E2, Canada

Due to a mistake in the publication process, “NS” symbols are missing from Figure 3 from this article [1]. The correct figure is shown below.

The change does not affect the scientific results. The manuscript will be updated and the original will remain online on the article webpage. We would like to apologize for any inconvenience caused to the readers by this mistake.

## Figures and Tables

**Figure 3:**
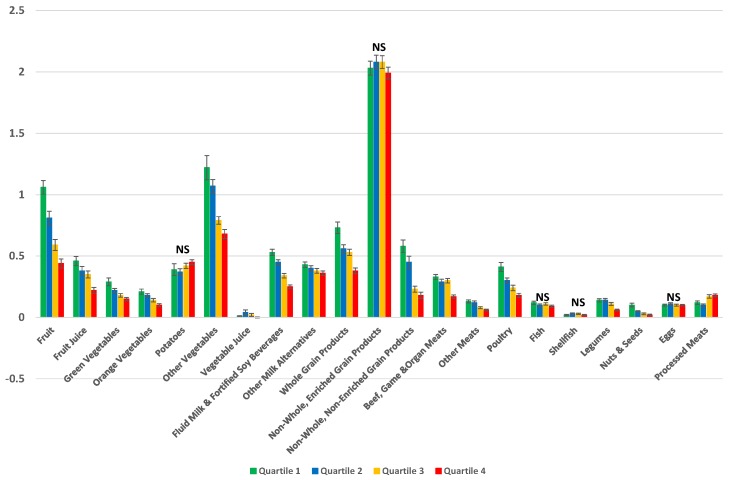
Implementation of 2014 Health Canada Surveillance Tool Tier system to the dietary intakes of Canadian adults (≥19 years) in a weighted analysis to assess the number of servings from each of the ‘Eating Well with Canada’s Food Guide’ subgroups per 1000 kcal [25]. Dietary profiles of compliers (Quartile 1) *, intermediates (Quartiles 2 and 3) ^†^, and non-compliers (Quartile 4) ^‡^ are compared ^§,‖^ NS, Not significant; * The 25% of individuals with the lowest percentage of energy from Tier 4 and “other” foods and beverages; ^†^ The individuals in the interquartile range for energy intakes from Tier 4 and “other” foods and beverages; ^‡^ The 25% of individuals with the highest percentage of energy from Tier 4 and “other” foods and beverages; ^§^ Adjusted for age, sex, and misreporting status (under-reporters, plausible-, and over-reporters); ^‖^ Quartiles are based upon percentage of energy from all Tier 4 foods based on 2014 Health Canada’s Surveillance Tool Tier system plus “other” foods and beverages not recommended in the Eating Well with Canada’s Food Guide.
