# Supplementary material for: Assessing the Nutritional Quality of Diets of Canadian Adults Using the 2014 Health Canada Surveillance Tool Tier System
Source: Nutrients. 2015 Dec 12;7(12):10447–68. doi: 10.3390/nu7125543 (PMC4690095; doi:10.3390/nu7125543)
Supplement: Supplementary file 1 [file nutrients-07-05543-s001.docx]

Supplementary Materials: Assessing the Nutritional Quality of Diets of Canadian Adults Using the 2014 Health Canada Surveillance Tool Tier System

Mahsa Jessri, Stephanie K. Nishi and Mary R. L’Abbé

**Table S1.** Initial placement of foods in Tiers using thresholds for fats, sodium and sugars.

| **Tiers** | **Fats, Sugars and Sodium Content of Foods** | | **Thresholds** |
| --- | --- | --- | --- |
| 1 | Foods that do not exceed any of the three lower thresholds for total fat, sugars and sodium * | | Lower thresholds: Total Fat: ≤3 g/RA Sugars: ≤6 g/RA Sodium: ≤140 mg/RA |
| 2 | Foods that exceed one or two lower thresholds for total fat, sugars or sodium, without exceeding any upper thresholds | |  |
| 3 | Vegetables and Fruit and Grain Products | Milk and Alternatives and Meat and Alternatives |  |
|  | Foods that exceed all three lower thresholds without exceeding any upper thresholds for total fat, saturated fat, sugars or sodium  OR  Foods that exceed only one upper threshold for total fat, saturated fat, sugars or sodium | Foods that exceed all three lower thresholds without exceeding any upper thresholds for total fat, sugars or sodium ^†^  OR  Foods that exceed only one upper threshold for total fat, sugars or sodium ^†^  OR  Foods that only exceed the upper saturated fat threshold |  |
| 4 | Vegetables and Fruit and Grain Products | Milk and Alternatives and Meat and Alternatives | Upper thresholds:  Total fat: >10 g/RA Sugars: >19 g/RA Sodium: >360 mg/RA Saturated fat: >2 g/RA |
|  | Foods that exceed at least two upper thresholds for total fat, saturated fat, sugars or sodium | Foods that exceed at least two upper thresholds for total fat, sugars or sodium ^†^ |  |

Source: ^©^ All Rights Reserved. The Development and Use of a Surveillance Tool: The Classification of Foods in the Canadian Nutrient File According to Eating Well with Canada’s Food Guide. Health Canada, 2014. Reproduced with permission from the Minister of Health, 2015. RA: Reference amount; * Can’t exceed the upper threshold for saturated fat; ^†^ Irrespective of saturated fat content (value may be above or below upper saturated fat threshold).

**Table S2.** Overview of food products reported in the Canadian Community Health Survey 2.2 based on the 2014 Health Canada’s Surveillance Tool Tier system [9] by food group.

| **Food Group by Tier** | **% within Corresponding Food Group/Category** | **% of Total Reported Food Products** |
| --- | --- | --- |
| **Tier 1 Foods** | | |
| Fruits & Vegetables Tier 1 | 76.11 | 18.57 |
| Grain Products Tier 1 | 19.09 | 2.99 |
| Milk & Alternatives Tier 1 | 15.56 | 1.89 |
| Meat & Alternatives Tier 1 | 7.52 | 0.77 |
| **Total Tier 1** |  | **24.22** |
| **Tier 2 Foods** | | |
| Fruits & Vegetables Tier 2 | 14.60 | 3.56 |
| Grain Products Tier 2 | 47.10 | 7.37 |
| Milk & Alternatives Tier 2 | 30.80 | 3.74 |
| Meat & Alternatives Tier 2 | 25.24 | 2.60 |
| **Total Tier 2** |  | **17.28** |
| **Tier 3 Foods** | | |
| Fruits & Vegetables Tier 3 | 6.14 | 1.50 |
| Grain Products Tier 3 | 18.87 | 2.95 |
| Milk & Alternatives Tier 3 | 39.21 | 4.76 |
| Meat & Alternatives Tier 3 | 51.11 | 5.26 |
| **Total Tier 3** |  | **14.48** |
| **Tier 4 Foods** | | |
| Fruits & Vegetables Tier 4 | 3.14 | 0.77 |
| Grain Products Tier 4 | 14.93 | 2.34 |
| Milk & Alternatives Tier 4 | 14.43 | 1.75 |
| Meat & Alternatives Tier 4 | 16.12 | 1.66 |
| **Total Tier 4** |  | **6.52** |
| **“Other” Foods and Beverages** | | |
| Unsaturated Fats and Oils | 10.1 | 3.75 |
| Saturated and/or  Trans Fats and Oils | 11.20 | 4.20 |
| Beverages, Higher Calorie  (>40 kcal/100 g) | 7.00 | 2.63 |
| Beverages, Lower Calorie  (<40 kcal/100 g) | 13.36 | 5.01 |
| Uncategorized (Ingredients/ seasonings, unprepared foods) | 31.26 | 11.73 |
| Alcoholic Beverages | 2.10 | 0.79 |
| High Fat and/or Sugar Foods | 23.77 | 8.92 |
| Meal Replacements | 0.16 | 0.06 |
| Supplements | 0.08 | 0.03 |
| Recipes | 0.22 | 0.08 |
| Foods and Beverages that are Not Classified | 0.83 | 0.31 |
| **Total “Other” Foods** |  | **37.50** |

**Table S3.** Overview of food products reported in the Canadian Community Health Survey 2.2 based on the 2014 Health Canada’s Surveillance Tool Tier system [9] by food sub-group *.

| **Food Sub-Group** | **Tier *** | **Absolute of Reported Food Products** | **% of Total Reported Products** | **% within Total Food Group** |
| --- | --- | --- | --- | --- |
| Fruit, other than juice | 1 | 51,291 | 7.51 | 19.24 |
|  | 2 | 1441 | 0.21 | 0.54 |
|  | 3 | 1006 | 0.15 | 0.38 |
|  | 4 | 31 | 0 | 0.01 |
| Fruit, juice | 1 | NA | NA | NA |
|  | 2 | 22,283 | 3.26 | 8.36 |
|  | 3 | 555 | 0.08 | 0.21 |
|  | 4 | NA | NA | 0.00 |
| **Total Fruit** |  | **76,607** | **11.22** | **28.74** |
| Vegetables, dark green | 1 | 21,141 | 3.10 | 7.93 |
|  | 2 | 1476 | 0.22 | 0.55 |
|  | 3 | 27 | 0.00 | 0.01 |
|  | 4 | NA | NA | 0.00 |
| Vegetables, deep yellow or orange | 1 | 17,217 | 2.52 | 6.46 |
|  | 2 | 300 | 0.04 | 0.11 |
|  | 3 | 204 | 0.03 | 0.08 |
|  | 4 | NA | NA | NA |
| Vegetables, potatoes | 1 | 12,731 | 1.86 | 4.78 |
|  | 2 | 2607 | 0.38 | 0.98 |
|  | 3 | 5361 | 0.79 | 2.01 |
|  | 4 | 8132 | 1.19 | 3.05 |
| Vegetables, other | 1 | 100,533 | 14.72 | 37.71 |
|  | 2 | 10,739 | 1.57 | 4.03 |
|  | 3 | 7883 | 1.15 | 2.96 |
|  | 4 | 220 | 0.03 | 0.08 |
| Vegetables, juice and cocktail | 1 | NA | NA | 0.00 |
|  | 2 | 89 | 0.01 | 0.03 |
|  | 3 | 1324 | 0.19 | 0.50 |
|  | 4 | NA | NA | NA |
| **Total Vegetable** |  | **189,984** | **27.82** | **71.26** |
| **Total Fruits & Vegetables** |  | **266,591** | **39.04** | **100** |
| Grain products, whole grain | 1 | 4396 | 0.64 | 2.57 |
|  | 2 | 19,397 | 2.84 | 11.34 |
|  | 3 | 3591 | 0.53 | 2.10 |
|  | 4 | 2350 | 0.34 | 1.37 |
| Grain products, non whole grain, enriched | 1 | 28,269 | 4.14 | 16.52 |
|  | 2 | 50,192 | 7.35 | 29.34 |
|  | 3 | 25,612 | 3.75 | 14.97 |
|  | 4 | 19,930 | 2.92 | 11.65 |
| Grain products, non whole grain, not enriched | 1 | NA | NA | 0.00 |
|  | 2 | 10,992 | 1.61 | 6.43 |
|  | 3 | 3085 | 0.45 | 1.80 |
|  | 4 | 3261 | 0.48 | 1.91 |

**Table S3.** *Cont.*

| **Food Sub-Group** | **Tier *** | **Absolute of Reported Food Products** | **% of Total Reported Products** | **% within Total Food Group** |
| --- | --- | --- | --- | --- |
| **Total Grain Products** |  | **171,075** | **25.05** | **100.00** |
| Fluid milk and fortified soy-based beverages | 1 | 20,521 | 3.00 | 15.46 |
|  | 2 | 39,377 | 5.77 | 29.67 |
|  | 3 | 13,908 | 2.04 | 10.48 |
|  | 4 | 428 | 0.06 | 0.32 |
| Other milk alternatives (cheese, yogourt) | 1 | 128 | 0.02 | 0.10 |
|  | 2 | 1503 | 0.22 | 1.13 |
|  | 3 | 38,130 | 5.58 | 28.73 |
|  | 4 | 18,721 | 2.74 | 14.11 |
| **Total Milk & Alternatives** |  | **132,716** | **19.43** | **100.00** |
| Beef, game and organ meats | 1 | 186 | 0.03 | 0.17 |
|  | 2 | 1924 | 0.28 | 1.71 |
|  | 3 | 19,644 | 2.88 | 17.45 |
|  | 4 | 149 | 0.02 | 0.13 |
| Other meats  (pork, veal, lamb) | 1 | 10 | 0 | 0.01 |
|  | 2 | 282 | 0.04 | 0.25 |
|  | 3 | 7523 | 1.10 | 6.68 |
|  | 4 | 49 | 0.01 | 0.04 |
| Poultry | 1 | 4341 | 0.64 | 3.86 |
|  | 2 | 4974 | 0.73 | 4.42 |
|  | 3 | 6592 | 0.97 | 5.86 |
|  | 4 | 2197 | 0.32 | 1.95 |
| Processed Meats | 1 | NA | NA | NA |
|  | 2 | 754 | 0.11 | 0.67 |
|  | 3 | 9259 | 1.36 | 8.23 |
|  | 4 | 13,981 | 2.05 | 12.42 |

**Table S3.** *Cont.*

| **Food Sub-Group** | **Tier *** | **Absolute of Reported Food Products** | **% of Total Reported Products** | **% within Total Food Group** |
| --- | --- | --- | --- | --- |
| **Total Meats** |  | **71,865** | **10.52** | **63.85** |
| Fish | 1 | 1891 | 0.28 | 1.68 |
|  | 2 | 2563 | 0.38 | 2.28 |
|  | 3 | 460 | 0.07 | 0.41 |
|  | 4 | 540 | 0.08 | 0.48 |
| Shellfish | 1 | 380 | 0.06 | 0.34 |
|  | 2 | 874 | 0.13 | 0.78 |
|  | 3 | 461 | 0.07 | 0.41 |
|  | 4 | 164 | 0.02 | 0.15 |
| Legumes | 1 | 1295 | 0.19 | 1.15 |
|  | 2 | 216 | 0.03 | 0.19 |
|  | 3 | 8895 | 1.30 | 7.90 |
|  | 4 | 881 | 0.13 | 0.78 |
| Nuts and seeds | 1 | 20 | 0.00 | 0.02 |
|  | 2 | 1070 | 0.16 | 0.95 |
|  | 3 | 3897 | 0.57 | 3.46 |
|  | 4 | 184 | 0.03 | 0.16 |
| Eggs | 1 | 344 | 0.05 | 0.31 |
|  | 2 | 15,750 | 2.31 | 13.99 |
|  | 3 | 796 | 0.12 | 0.71 |
|  | 4 | NA | NA | 0.00 |
| **Total Meat Alternatives** |  | **40,681** | **5.96** | **36.15** |
| **Total Meat & Alternatives** |  | **112,546** | **16.48** | **100** |

* Tiers are based on Health Canada’s Surveillance Tool and defined generally as follows: Tier 1–3 foods are compliant with EWCFG and Tier 4 foods are not recommended by the EWCFG. Tier 1 foods are foods that do not exceed lower thresholds for total fat, sugars, and sodium; Tier 2 foods do not exceed up to 2 lower thresholds for total fat, sugars or sodium, without exceeding any upper thresholds; for the Vegetables and Fruit and Grain Products food groups Tier 3 are foods that exceed all 3 lower thresholds without exceeding any upper thresholds or exceed only one upper threshold, while Tier 4 foods exceed at least 2 upper thresholds for total fat, saturated fat, sugars, or sodium. Within the Milk and Alternatives and Meat and Alternatives food groups, Tier 3 foods exceed all 3 lower thresholds without exceeding any upper thresholds for total fat, sugars, or sodium (irrespective of saturated fat) or exceed only one of these 3 thresholds or foods that only exceed the upper saturated fat threshold; within these 2 food groups foods that exceed at least 2 upper thresholds for total fat, sugars, or sodium were classified as Tier 4. Where lower thresholds entail: total fat < 3 g/RA, sugars < 6 g/RA, and sodium <140 mg/RA; and upper thresholds are: total fat
>10 g/RA, sugars >19 g/RA, sodium >360 mg/RA, and saturated fat >2 g/RA. Full details are shown in Table S1.

**Table S4.** Weighted regression analysis of the association between quartiles of the percent energy from Tier 4 and “other” foods/beverages and risk of obesity in Canadian adults (≥19 years) *.

|  | **Compliers (Q1) ^†^ ≤19.42% Energy** | **Intermediates (Q2) ^‡^** **19.42%–31.78% Energy** | **Intermediates (Q3) ^‡^** **31.78%–45.73% Energy** | **Non-Compliers (Q4) ^§^ >45.73% Energy** |  |
| --- | --- | --- | --- | --- | --- |
| **Characteristics** | **OR (95%CI)** | **OR (95%CI)** | **OR (95%CI))** | **OR (95%CI)** | ***p*-Trend** |
| **Obesity, (BMI ≥30 kg/m^2^)** | | | | | |
| Model 1 ^‖^ | 1.0 | 0.872 (0.646–1.176) | 1.047 (0.792–1.384) | 1.058 (0.799–1.397) | 0.7053 |
| Model 2 ^¶^ | 1.0 | 0.912 (0.675–1.233) | 1.815 (0.894–1.571) | 1.294 (0.971–1.724) | 0.0657 |

95% CI: 95% Confidence Interval; BMI, Body Mass Index; OR, Odds Ratio; * Quartiles are based upon percentage of energy from all Tier 4 foods based on 2014 Health Canada’s Surveillance Tool Tier system [9] plus “other” foods and beverages not recommended in the Eating Well with Canada’s Food Guide; ^†^ The 25% of individuals with the lowest percentage of energy from Tier 4 and “other” foods and beverages; ^‡^ The individuals in the interquartile range for energy intakes from Tier 4 and “other” foods and beverages; ^§^ The 25% of individuals with the highest percentage of energy from Tier 4 and “other” foods and beverages; ^‖^ Adjusted for age and sex; ^¶^ Adjusted for age and sex and misreporting status (under-reporter, plausible-, and over-reporters).
